# *Ulva lactuca* Extract in Confectionery Systems: A Sustainable Approach to Natural Pigmentation, Antioxidant Enrichment, and Sensory Optimization in Fondant and Meringue Matrices

**DOI:** 10.1002/fsn3.72079

**Published:** 2026-07-02

**Authors:** Elvan Gizem Gursoy, Buse Ozdere Yilmaz, Aysegul Erdogan, Meltem Conk Dalay, Sebnem Tavman

**Affiliations:** ^1^ Department of Food Engineering Ege University Izmir Turkey; ^2^ Central Research Test and Analysis Laboratory Application and Research Center Ege University Izmir Turkey; ^3^ Department of Bioengineering Ege University Izmir Turkey

**Keywords:** antioxidant, confectionery, functional foods, natural colorant, *Ulva*

## Abstract

The replacement of synthetic additives with sustainable, marine‐derived bioactives represents a critical step toward meeting the growing consumer demand for clean‐label and functional confectionery products. This study aimed to investigate the functional potential of *Ulva lactuca* extract as a natural colorant and antioxidant in sugar‐based confectionery (fondant) and aerated desserts (meringue). The green macroalga, rich in phenolic compounds and pigments, was incorporated at different concentrations to assess its impact on chromatic properties, antioxidant capacity, physicochemical stability, texture, and sensory acceptability. Comprehensive analyses included CIE L*a*b* colorimetry, dry matter content, water activity, DPPH/ABTS radical scavenging assays, total phenolic quantification, FT‐IR, SEM, texture profile analysis, and hedonic sensory evaluation. *Ulva* extract significantly enhanced total phenolic content and antioxidant activity for both model matrices in a dose‐dependent manner. The meringue matrix showed a ≈7‐fold increase in phenolic levels, reaching up to 150.95 mg GAE/100 g dry matter at 0.63% extract, while maximum antioxidant capacities achieved were 33.42 μM TEAC/g dry matter and 53.28 μM TEAC/g dry matter for ABTS and DPPH assays, respectively. Chromatic shifts toward green‐yellow hues aligned with chlorophyll and carotenoid profiles, while textural analysis revealed significant alterations. Sensory evaluation identified matrix‐specific optimal thresholds, with fondants demonstrating higher resilience (up to 0.31%) compared to meringues (0.08%–0.16%) in balancing enrichment with acceptability. Overall, meringues exhibited superior functional performance, achieving a substantially higher phenolic fortification and antioxidant surge compared to the fondant matrix. These findings position *Ulva lactuca* extract as a sustainable, multifunctional ingredient for sugar‐based confections, offering antioxidant fortification and natural pigmentation. The study underscores the importance of concentration thresholds to harmonize functional benefits with sensory quality, advancing the development of clean‐label, marine‐derived functional foods.

## Graphical abstract

This study evaluated the functional potential of Ulva lactuca extract as a natural colorant and antioxidant incorporated into sugar‐based (fondant) and aerated (meringue) confectionery matrices. Extract incorporation significantly enriched total phenolic content—achieving up to 150.95 mg GAE/100 g dry matter in meringues—and surged antioxidant capacities across ABTS and DPPH assays. Chromatic shifts toward green‐yellow hues aligned with algal pigment profiles, while structural analyses revealed dose‐dependent alterations in matrix integrity. Sensory evaluation identified optimal inclusion thresholds (0.31% for fondant and 0.16% for meringue), demonstrating this sustainable, marine‐derived strategy effectively harmonizes functional fortification with clean‐label sensory quality.

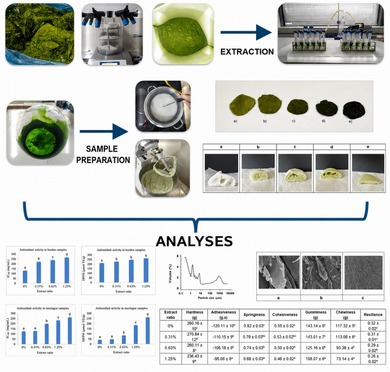

## 1 Introduction

Growing health concerns regarding synthetic colorants have accelerated the search for natural pigments (Gebhardt et al. 2020). Synthetic additives face scrutiny due to potential health and ecological risks, driving exploration of bioactive‐rich natural sources (Novais et al. 2022). This transition is further accelerated by the current regulatory landscape in both the European Union and the USA, where recent legislative trends increasingly limit the use of synthetic food dyes in favor of plant‐ and marine‐derived alternatives (FDA 2025). In the EU, such natural alternatives are highly favored as they can be classified as “coloring foodstuffs” rather than food additives, circumventing stringent E‐number regulations and aligning with clean‐label demands (Herrera et al. 2023). Recent studies demonstrate the potential of plant and algal extracts, such as aronia (Ghendov‐Mosanu et al. 2022), barberry (Çoban et al. 2021), carob (Ibrahim et al. 2020), coffee exocarp (Parra‐Campos and Ordóñez‐Santos 2019), and green macroalgae like *Ulva lactuca* (Jayasinghe et al. 2016). Among these, marine macroalgae have emerged as promising candidates, offering a reservoir of phytochemicals with multifunctional properties. *Ulva* sp., noted for rapid growth, ease of cultivation, and richness in phenolic compounds, pigments, and sulfated polysaccharides like ulvan (Yildiz et al. 2012). Beyond these bioactives, this species is characterized by a high mineral content, which may impart a distinct saline or marine flavor profile to the final product. Such organoleptic characteristics are critical factors that can potentially influence sensory acceptance and consumer perception, particularly in sugar‐based confectionery systems (Khan et al. 2024).

Furthermore, the commercial integration of Ulva species into the food industry must comply with strict regulatory frameworks. Although *Ulva lactuca* has a documented history of consumption and is generally not classified as a Novel Food in the EU, the application of its concentrated extracts must align with the European Food Safety Authority (EFSA) guidelines. These regulations emphasize the necessity of monitoring safe intake limits for potential hazards, such as iodine and heavy metal accumulation, ensuring that such functional extracts meet rigorous consumer safety standards and compliance criteria for “coloring foodstuffs” (EFSA 2023; European Union 2015). The primary objective of this study was to evaluate the functional, chromatic, and structural applicability of *Ulva lactuca* extracts in model confectionery matrices. Therefore, the quantitative determination of iodine and heavy metal contents was considered beyond the scope of the present investigation. Nevertheless, a comprehensive assessment of these safety parameters, including the quantification of iodine and heavy metal levels, will be essential for future commercial applications and for the development of products that comply with relevant regulatory requirements.

Confectionery products, particularly sugar‐based systems like fondant and meringue, typically lack functional components and rely on synthetic additives for color and shelf‐life (Korkach and Krusir 2017). Reformulating these matrices to incorporate natural bioactive compounds could enhance their nutritional profile while aligning with clean‐label trends. However, such modifications pose challenges, as the introduction of bioactive ingredients may alter physicochemical properties, texture, and sensory acceptability (Tolve and Simonato 2024). For instance, phenolic compounds can interact with proteins in aerated matrices like meringues, destabilizing foam structures and compromising texture (Ozdal et al. 2013). Thus, balancing functional enrichment with structural and sensory integrity remains a critical hurdle.

Antioxidants, defined as compounds capable of neutralizing free radicals, play a vital role in mitigating oxidative damage linked to chronic conditions such as diabetes, atherosclerosis, and neurodegenerative disorders (Pai et al. 2022; Lobo et al. 2010). Marine algae, particularly green macroalgae like *Ulva* sp., are rich in bioactive pigments such as carotenoids and chlorophylls, which have garnered increasing interest as natural antioxidant sources (El‐Beltagi et al. 2022). Carotenoids, widely studied for their health‐promoting effects, contribute to cognitive preservation, skin health, and inflammation reduction, primarily through their radical‐scavenging activity (Tiwari et al. 2022; Imchen and Singh 2023). Chlorophylls, abundant in *Ulva*, demonstrate comparable antioxidant efficacy via derivatives like pheophorbides, which exhibit DPPH and hydroxyl radical scavenging capacities (Tumolo and Lanfer‐Marquez 2012; Chen and Roca 2018; Cho et al. 2011).

There is growing evidence for the successful integration of algal bioactives into food matrices to enhance antioxidant properties. For instance, microalgae (*Arthrospira platensis*, *Chlorella vulgaris* , *Tetraselmis suecica*, and *Phaeodactylum tricornutum* ) elevated antioxidant activity in wheat‐based crackers dose‐dependently (Batista et al. 2019). Similarly, *Ulva lactuca* and *Ulva fasciata* (4% w/w) enhanced DPPH scavenging in crackers by 41.09%–42.30% respectively, compared to controls (Egodavitharana et al. 2023). Biscuits enriched with 5% *Caulerpa racemosa* showed superior antioxidant performance (Kumar et al. 2018). Extruded snacks with *Fucus vesiculosus* extract (2%–5%) retained bioactivity post‐processing, achieving TEAC values > 10 μmol/g without compromising sensory appeal (Corsetto et al. 2020). Non‐algal ingredients, such as pumpkin flour (20% substitution in bread), also enhanced antioxidant capacity (DPPH: 76.59%; ABTS: 81.74%) and phenolic content (5.39 mg GAE/g), underscoring the broader applicability of natural additives (Wahyono et al. 2020).

Phenolic compounds, central to the antioxidant function of natural colorants like *Ulva* extracts, play a key role in their functional potential (Cappa et al. 2015). Beyond their technological applications, marine polyphenols offer profound human health benefits by mitigating cellular oxidative stress. Their dietary intake is strongly linked to significant anti‐inflammatory, neuroprotective, and cardioprotective effects, positioning them as key bioactive agents in the prevention of chronic non‐communicable diseases. *Ulva* species, rich in phenolic compounds and pigments, are promising candidates for functional food additives, with phenolic content varying by cultivation conditions and extraction methods (Ning et al. 2022). Ethanol‐based extraction (95% v/v) yields the highest phenolic content (1.58 mg GAE/g dry weight), while naturally grown *Ulva* may contain up to 4600 μg/mL of phenolics (Abd El‐Baky et al. 2009; McCauley et al. 2018). Despite the recognized bioactive potential of marine macroalgae, their incorporation into confectionery products remains limited due to insufficient knowledge regarding the behavior and stability of marine‐derived bioactive compounds under the unique physicochemical conditions of confectionery systems, including high osmotic pressure, sugar crystallization, and protein‐foam destabilization. This study investigates the use of *Ulva lactuca* extract as a multifunctional ingredient for the development of clean‐label, bioactive‐enriched confections while maintaining essential textural and sensory qualities. Fondant and meringue were selected as model systems due to their contrasting physicochemical architectures: fondant is a dense, saturated sugar‐crystal matrix, whereas meringue is a sensitive, aerated protein foam. The behavior and pigment stability of chlorophyll‐rich seaweed extracts within these distinct environments remain underexplored. Furthermore, these systems allow for a comparative evaluation of processing impacts—specifically, the addition of the extract post‐thermal treatment in fondant versus pre‐baking in meringue—on the stability and extractability of marine bioactives.

Beyond simple ingredient incorporation, this study aims to develop a functional and sustainable confectionery system by integrating *Ulva lactuca* extract into fondant and meringue matrices, exploring the interplay between nutritional fortification and technological performance (encompassing textural attributes and physicochemical properties such as dry matter and water activity). Therefore, the primary objective of this research is to comprehensively evaluate the dose‐dependent effects of the extract on phenolic enrichment, physicochemical properties, and textural integrity, ultimately establishing optimal incorporation thresholds that balance functional fortification with consumer acceptability. By integrating these technological and nutritional aspects, this study provides a practical approach for clean‐label confectionery development.

## 2 Materials and Methods

### 2.1 Materials and Equipment

**FIGURE 1:**
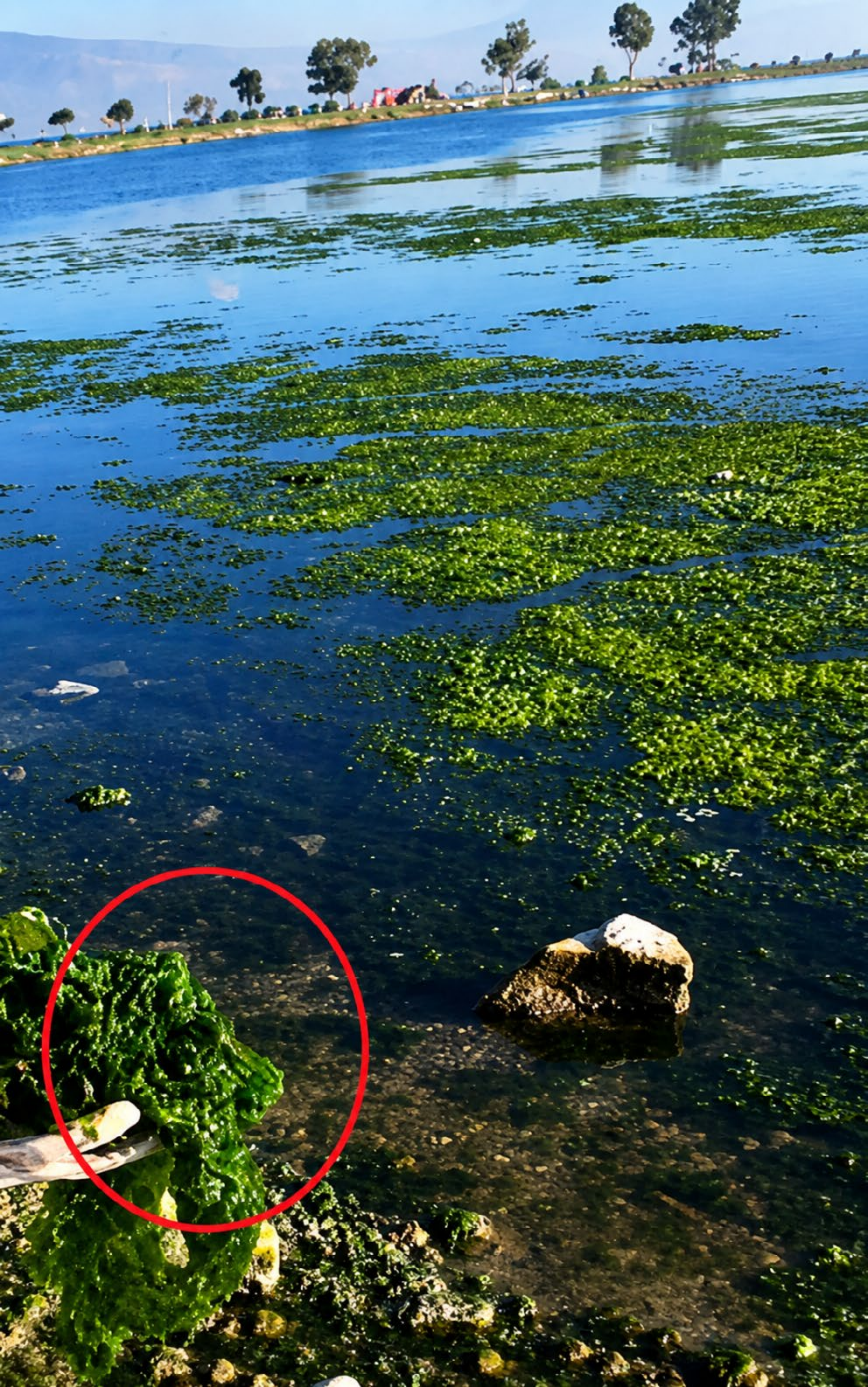
*Ulva lactuca* samples collected from Cakalburnu Lagoon, Izmir, Türkiye.

*Ulva lactuca* samples were collected from Cakalburnu Lagoon, southern Izmir Bay, Türkiye (38°24′45″ N 27°02′54″ E) during the seasonal bloom period in January 2024 (Figure 1). Fresh thalli were thoroughly rinsed under running tap water to remove adherent sand, stones, and epibionts, then freeze‐dried (until a constant weight was achieved to protect thermolabile bioactives), pulverized, and stored at −18°C in sealed plastic bags (Turner 2022).

Fondant and meringue ingredients included locally sourced glucose syrup, icing sugar (confectioner's sugar), and spray‐dried egg white powder procured from Tunalı Grup Gıda Koz. Ltd. Şti., Ankara.

Chemicals used: (2,2′‐azino‐bis(3‐ethylbenzothiazoline‐6‐sulfonic acid diammonium salt)), DPPH (2,2‐diphenyl‐1‐picrylhydrazyl), Trolox (6‐hydroxy‐2,5,7,8‐tetramethylchroman‐2‐carboxylic acid), pyrogallol (Sigma Aldrich, St. Louis, Missouri, USA), calcium carbonate, sodium carbonate, Folin–Ciocalteu reagent, gallic acid, potassium persulfate, methanol, ethanol (Merck, Darmstadt, Germany). All chemicals and solvents used in the spectrophotometric analyses were of analytical reagent grade.

The primary analytical instruments and equipment utilized throughout this study included an ultrasonic bath (Elmasonic S80H, Elma Schmidbauer GmbH, Germany), a centrifuge (Hettich Universal 320R cooled, Germany), a laboratory freeze‐dryer (Alpha 1–2 LDplus, Martin Christ, Germany), a rotary evaporator (Stuart RE 400, Cole‐Parmer, UK), a texture analyzer (TA.XT2i, Stable Micro Systems, UK), a UV–Vis spectrophotometer (Cary 60, Agilent Technologies, Santa Clara, CA, USA), a hand spectrophotometer (CM‐700D, Konica Minolta Sensing, Japan), a Testo AG400 water activity analyzer (Testo AG, Germany), a digital caliper (Mitutoyo Digimatic Caliper, Model CD‐20APX, Japan), a homogenizer (IKA Ultraturrax T 25 D, Germany), a vacuum oven (WiseVen WOW‐30, Daihan Scientific, Seoul, Republic of Korea), a baking oven (Vestel, Pyro BO67, Türkiye), an FT‐IR spectral analysis instrument (Thermo Scientific NICOLET iS10, USA), and a scanning electron microscope (ThermoFisher QUANTA FEG 250, USA).

### 2.2 Extraction of Bioactive Compounds From *Ulva lactuca*

Ultrasound‐assisted extraction (UAE) was performed using an Elmasonic S80H ultrasonic bath (Elma Schmidbauer GmbH, Germany; 37 kHz, 150 W effective power), as a widely used technique for efficient recovery of bioactive compounds from marine algae due to its enhanced mass transfer and reduced processing time (Kadam et al. 2013). The extraction conditions (40°C for 30 min with a biomass‐to‐solvent ratio of 0.05 g/mL) were established based on preliminary laboratory trials. These specific parameters were selected to maximize the recovery of targeted bioactives while actively preventing the thermal degradation of sensitive pigments. The biomass was subsequently frozen at −20°C and lyophilized for 48 h using a freeze‐dryer (Alpha 1–2 LDplus, Martin Christ, Osterode am Harz, Germany). This vacuum‐assisted low‐temperature drying process was preferred to ensure the maximum retention of thermolabile pigments (chlorophylls and carotenoids) and polyphenolic compounds present in the *Ulva lactuca* matrix. Dried *Ulva lactuca* biomass was accurately weighed, combined with 0.5 g CaCO_3_ to minimize pigment degradation, and suspended in 10.0 mL absolute ethanol (100%) containing 0.01% (w/v) pyrogallol, a known antioxidant agent used to prevent oxidation of sensitive pigments such as carotenoids and chlorophylls. After sonication under the aforementioned conditions, the suspension was centrifuged (5000 rpm, 5 min). The supernatant was collected, and the solid residue underwent sequential re‐extractions with fresh ethanol until visual decolorization. Pooled supernatants were subsequently vacuum‐filtered (nylon membrane, diameter: 47 mm, pore size: 0.20 μm), then concentrated via rotary evaporation (Stuart RE 400, Cole‐Parmer, UK; 40°C, 400 mbar). Quantitative results of the extraction yielded: total carotenoids 0.235 mg/g, chlorophyll a* 0.849 mg/g, chlorophyll b* 1.756 mg/g, respectively, under these conditions.

### 2.3 Determination of Chlorophyll a, Chlorophyll b, and Total Carotenoids

The chlorophyll and carotenoid contents of *Ulva lactuca* extracts were determined spectrophotometrically following the method described by Dere et al. (1998). The spectrophotometric approach was selected due to its simplicity, rapidity, and suitability for the quantitative determination of photosynthetic pigments in green macroalgae. Ethanolic extracts obtained after the extraction procedure were used directly for pigment analysis. Absorbance measurements were carried out using a UV–Vis spectrophotometer at wavelengths of 666, 653, and 470 nm, corresponding to the absorption maxima of chlorophyll a, chlorophyll b, and total carotenoids, respectively. The concentrations of chlorophyll a (*C*_a_) and chlorophyll b (*C*_b_) were calculated from the absorbance values measured at 666 and 653 nm using the following equations:

$$C_{a}\;\left(\mathrm{mg}\;{\mathrm{mL}}^{-1}\right)=15.65\times A666-7.340\times A653$$

$$C_{b}\;\left(\mathrm{mg}\;{\mathrm{mL}}^{-1}\right)=27.05\times A653-11.21\times A666$$

Total carotenoid content (*C*_x+c_) was subsequently calculated using the absorbance measured at 470 nm and the previously determined chlorophyll concentrations:

$$C_{x+c}\;\left(\mathrm{mg}\;{\mathrm{mL}}^{-1}\right)=\left(1000\times A470–2.860\times C_{a}-129.2\times C_{b}\right)/245$$

where A666, A653, and A470 denote the absorbance values recorded. These equations compensate for the spectral overlap between chlorophylls and carotenoids, thereby providing a more accurate estimation of individual pigment concentrations in the ethanolic extracts.

Pigment concentrations were initially expressed as mg mL^−1^ extract and subsequently converted to mg g^−1^ dry weight (DW) of *Ulva lactuca* biomass by considering final extract volume and dry biomass used during extraction.

### 2.4 Preparation of Fondant and Meringue Samples

Fondant samples were prepared using icing sugar, modifying the recipe from the study by Edwards (2009). Briefly, the fondant base was prepared by boiling the sugar mixture to 114°C, followed by cooling to 49°C and mixing for 10–12 min to obtain a thick, opaque crystalline structure. To minimize the thermal degradation of heat‐sensitive pigments, the *Ulva lactuca* extract was incorporated into the fondant matrix at the final stage of processing, when the temperature had decreased to approximately 40°C. The extract quantities determined through preliminary trials conducted following a literature review. To evaluate the coloring efficacy of *Ulva lactuca* extract within the fondant matrix, the extract was incorporated into the prepared fondant at concentrations of 1.25%, 0.63%, 0.31%, and 0% (w/w). The formulation for the control sample is provided in Table 1.

**TABLE 1:** Fondant formulation (weight %).

Ingredient	Ratio (%)
Icing sugar	74
Glucose syrup	14.6
Water	11.4

**FIGURE 2:**
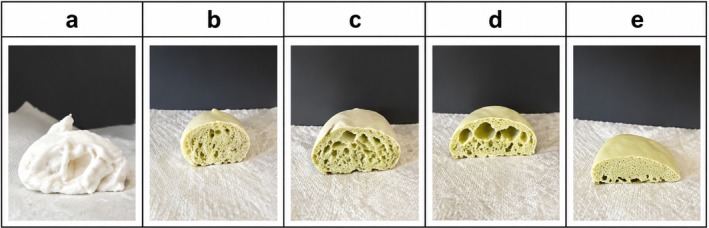
Meringue samples with different ratios of *Ulva lactuca* extract: (a) 0% (control), (b) 0.08% extract, (c) 0.16% extract, (d) 0.31% extract, and (e) 0.63% extract.

The preparation of meringues was adapted from the methodology by Wouters et al. (2018). To assess the coloring potential of *Ulva lactuca* extract, it was added to the meringue foam at concentrations of 0.63%, 0.31%, 0.16%, 0.08%, and 0% (w/w). The meringue foam mixtures were portioned into circular aliquots and spooned onto parchment paper. The samples were subsequently baked in a convection oven (Vestel, Pyro BO67, Türkiye) using upper and lower heating elements with fan‐assisted air circulation at 105°C for 90 min. Following baking, the samples were gradually cooled inside the oven at 50°C for 180 min. Baked meringue samples with different extract ratios are shown in Figure 2. Prepared samples were stored in sealed plastic bags under refrigeration (4°C) until analyses. The formulation for the control sample is detailed in Table 2.

**TABLE 2:** Meringue formulation (weight %).

Ingredient	Ratio (%)
Icing sugar	68.4
Water	27.4
Egg white powder	4.2

Analyses were conducted on fondant samples and meringues colored using the extract, as well as on control samples without the extract.

### 2.5 Color Analysis of Fondant and Meringue Samples

Color measurements were performed using a Konica Minolta spectrophotometer (CM‐700D, Konica Minolta Sensing, Japan) based on the CIE L*a*b* color space, which is widely used for objective color quantification in food systems (Pathare et al. 2013). The L* value represents lightness (0 = black, 100 = white), a* denotes the red‐green axis, and b* indicates the yellow‐blue axis. Measurements were recorded for 3 samples and a minimum of eight measurements were taken per sample, and was compared to the control. Differences in L*, a* and b* values were used to calculate the total color difference (ΔE*).

### 2.6 Dry Matter Content

The dry matter contents of fondant and meringue samples were determined using a modified method based on Troller and Christian (1978), with adjustments to drying duration and vacuum parameters. 5 g aliquots of 3 samples for each product were dried in a vacuum oven (WiseVen WOW‐30, Daihan Scientific, Seoul, Republic of Korea) at 70°C under a vacuum of −400 mmHg.

The moisture content values obtained from this vacuum‐drying process were utilized to normalize all biochemical analysis results (TPC, DPPH, and ABTS), ensuring that data are expressed on a dry weight basis (e.g., mg GAE/100 g dry sample) to account for moisture variations between the matrices.

### 2.7 Water Activity

The water activity (*a*_w_) of fondant and meringue samples was measured in triplicate using a Testo AG400 water activity analyzer (Testo AG, Germany), as outlined by Červenka et al. (2006). Prior to analysis, meringue samples were pulverized with a mortar and pestle. For measurement, the sample was placed in a sealed stainless steel chamber, and the system was allowed to equilibrate until the relative humidity of the headspace stabilized. The equilibrium moisture value was directly measured using an integrated probe within the chamber.

### 2.8 Preparation of Fondant and Meringue Extracts

For fondant extracts, 10 mL of 80% (v/v) methanol was added to 2 g of sample. The mixture was homogenized using a magnetic stirrer operating at 100 rpm (23 h, room temperature), then held at 40°C for 10 min. After centrifugation (10,000 × *g*, 10 min, 4°C), the supernatant was collected for analysis. Similarly, for control and *Ulva*‐incorporated meringue samples, 10 mL of 80% (v/v) methanol was added to 2.5 g of sample. Homogenization used an IKA Ultraturrax T 25 D (Germany) at speed level 7 (1 min, room temperature). After centrifugation (10,000 × *g*, 20 min, 4°C), the supernatant was collected (O'Charoen et al. 2014).

### 2.9 Total Phenolic Content in Sample Extracts

The total phenolic content in extracts derived from fondant and meringue samples was quantified in triplicate according to the Folin–Ciocalteu method described by Singleton et al. (1999). Absorbance was measured at 750 nm using a Cary 60 UV–Vis spectrophotometer (Agilent Technologies, Santa Clara, CA, USA). Results were expressed as milligrams of gallic acid equivalent (GAE) per 100 g of dry sample (mg GAE/100 g).

### 2.10 Antioxidant Activity by DPPH Assay

The antioxidant activity of samples was evaluated in triplicate via the DPPH (2,2‐diphenyl‐1‐picrylhydrazyl) radical scavenging assay, following the protocol outlined by Brand‐Williams et al. (1995). This widely used method is based on the reduction of the stable DPPH radical by antioxidants through a redox reaction (Lourenço‐Lopes et al. 2021).

For the assay, DPPH working solution was prepared with 80% (v/v) methanol. Aliquots of extracts (prepared from fondant or meringue samples) were mixed with DPPH radical solution to create test mixtures for both control samples and samples containing varying concentrations of *Ulva lactuca* extract. After incubation in the dark at room temperature, absorbance values were measured at 515 nm using a Cary 60 UV–Vis spectrophotometer (Agilent Technologies, Santa Clara, CA, USA). The antioxidant capacity was quantified by comparing a Trolox standard curve generated from known concentrations of Trolox. Results were expressed as micromolar Trolox equivalent antioxidant capacity (μM TEAC/g dry matter).

### 2.11 Antioxidant Activity by ABTS Assay

The ABTS [2,2′‐azino‐bis(3‐ethylbenzothiazoline‐6‐sulfonic acid)] radical scavenging assay was performed in triplicate according to the method described by Re et al. (1999). For the assay, the ABTS stock solution was combined with a potassium persulfate (K_2_S_2_O_8_) solution and allowed to stand in the dark at room temperature for 16 h to generate the ABTS radical cation (ABTS^•+^). Prior to analysis, the absorbance of the ABTS solution was adjusted to 0.7 at 734 nm using methanol.

For testing, diluted extracts (prepared from fondant or meringue samples) were mixed with the adjusted ABTS solution. After incubation in the dark at room temperature, absorbance was measured at 734 nm using a Cary 60 UV–Vis spectrophotometer (Agilent Technologies, Santa Clara, CA, USA). Results were expressed as micromolar Trolox equivalent antioxidant capacity (μM TEAC/g dry matter) based on a standard curve of Trolox.

### 2.12 Baking Yield

The baking yield of meringue samples was determined in triplicate, according to the method described by Altunakar (2003). Baking yield was calculated by dividing the post‐baking weight of the meringue samples (*W*_sample_) by the pre‐baking weight of the meringue foam (*W*_foam_) and computed using:

(1) $$\text{Baking Yield}\%=\left[\frac{\text{Post}-\text{baking weight}\;\left(g\right)}{\mathrm{Pre}-\text{baking weight}\;\left(g\right)}\right]\times 100$$

### 2.13 Volume Index

The volume index of meringue samples was determined in triplicate using AACC Method 10–91 (AACC 1983). Following this method, meringue samples were bisected vertically through the center, and the heights at three distinct points (*B*, *C*, and *D*) along the cross‐sectional area were measured using a digital caliper (Mitutoyo Digimatic Caliper, Model CD‐20APX, Japan). The volume index was calculated by:

(2) Volume Index=B×C+D

where *C* represents the central height, and *B* and *D* correspond to the heights measured at points located five‐thirds of the distance from the center to the edge.

### 2.14 Texture Profile Analysis

For fondant samples, the textural parameters were measured by using the TA.XT2i Texture Analyzer (Stable Micro Systems, Surrey, UK) equipped with a 5 kg load cell. Measurements were recorded for 3 samples, and a minimum of five measurements were taken per sample. Each sample was cut as a cylindrical solid and compressed under a cylindrical probe (P/25) with a 25% strain level at 2 mm/s test speed (Ozcan et al. 2019).

The textural properties of meringue samples were analyzed using a TA.XT2i Texture Analyzer (Stable Micro Systems, Surrey, UK) equipped with a 36‐mm cylindrical probe (P/36R). Testing parameters included a crosshead speed of 2 mm/s, compression to 75% of the original sample thickness, and a 2‐s interval between consecutive compression cycles. Measurements were recorded for 3 samples, and a minimum of five measurements were recorded per sample to ensure reproducibility, following the methodology outlined by Yüceer and Asik (2020).

### 2.15 Sensory Evaluation

The sensory evaluation of fondant and baked meringue samples was performed by a semi‐trained panel of 15 assessors using a scoring test. Panelists were recruited from the graduate students and faculty members of Ege University Department of Food Engineering. The samples were assessed for appearance, texture, odor, and overall acceptability using a 9‐point hedonic scale (1 = *dislike extremely*, 9 = *like extremely*), as outlined by Yüceer and Caner (2021). Before evaluation, each panelist was briefed about the product, the attributes to be assessed, and the use of the hedonic scale. Panelists worked in a sensory laboratory in individual sensory booths. Samples were labeled with three randomly chosen numbers and drinking water for palate cleansing.

### 2.16 Structural Characterization via FT‐IR Spectroscopy

FT‐IR spectral analysis was performed on dry *Ulva lactuca* samples using a Thermo Scientific NICOLET iS10 (USA) instrument within the wavenumber range of 400–4000 cm^−1^ at Ege University Central Research Test and Analysis Laboratory Application and Research Center (EGE MATAL).

### 2.17 Microstructural Analysis Using Scanning Electron Microscopy (SEM)

The morphological characteristics of *Ulva lactuca* were observed using a scanning electron microscope (ThermoFisher QUANTA FEG 250, USA) at Ege University Central Research Test and Analysis Laboratory Application and Research Center (EGE MATAL). Samples were coated with a conductive gold–palladium layer prior to imaging. Observations were conducted at 65,000×, 20,000×, and 2000× magnification.

### 2.18 Statistical Analysis

Statistical analysis was performed using SPSS 25.0 (IBM Corp., Armonk, NY). All analyses were carried out on three independent samples. Each sample was measured with appropriate technical replicates depending on the method. Differences between samples based on *Ulva* extract content were assessed by one‐way analysis of variance (ANOVA), with Duncan's test applied to determine group differences for significant results. A significance level of *p* < 0.05 was adopted for all analyses. Results are expressed as mean ± standard deviation. Relevant curves were generated using Microsoft Office Excel 2019.

## 3 Results and Discussion

### 3.1 Color Analysis of Fondant and Meringue Samples

The control fondant (0% extract) exhibited high lightness (L* = 88.99 ± 0.23), near‐neutral green‐red coordinate (a* = −0.23 ± 0.01), and low yellowness (b* = 6.46 ± 0.07), consistent with an off‐white appearance. Increasing *Ulva* extract concentrations (0.31%–1.25% w/w) induced significant (*p* < 0.05) alterations in color parameters compared to the control. At 0.31% extract, lightness (L*) decreased substantially to 60.05 ± 0.40, greenness (a*) increased significantly to −4.42 ± 0.05, and yellowness (b*) rose pronouncedly to 18.62 ± 0.29, yielding a large total color difference (∆E* = 31.67 ± 4.61). Further concentration increases caused significant, concentration‐dependent changes: L* decreased progressively (55.12 ± 0.59 at 0.63%; 49.04 ± 0.12 at 1.25%); greenness intensified significantly from −4.42 ± 0.05 (0.31%) to −4.62 ± 0.04 (0.63%), but shifted significantly less negative at 1.25% (−3.85 ± 0.04) compared to the 0.63% sample, indicating a reduction in greenness intensity. Yellowness (b*) increased significantly from 18.62 ± 0.29 (0.31%) to 19.78 ± 0.31 (0.63%) before decreasing significantly to 16.57 ± 0.31 (1.25%). Consequently, ∆E* increased significantly with each increment in extract concentration: 31.67 ± 4.61 (0.31%), 36.65 ± 4.88 (0.63%), and 41.36 ± 3.52 (1.25%).

After one month of storage, color stability was best preserved at 0.31% *Ulva*, showing minimal fluctuations in a* and b* values despite notable L* alterations. This aligns with prior observations in jelly matrices colored with *Rhoeo spathacea* (Swartz) extract, where L* variability was linked to physical matrix changes (e.g., surface roughness), while a* and b* stability suggested chromophore resilience (Tan et al. 2014). Similarly, roselle ( *Hibiscus sabdariffa* ) anthocyanins exhibited lightness and chroma shifts during storage. This phenomenon was attributed to anthocyanin degradation over storage time, leading to a dulling or loss of vibrancy in the samples' color, underscoring the broader challenge of balancing chromatic fidelity with environmental susceptibility in natural colorants (Duangmal et al. 2008). Color analysis results of fondants (initial and after 1 month storage) and percentage change rates are presented in Tables 3, 4, 5, respectively. From a practical standpoint, changes below 5% are generally considered below the threshold of human sensory perception and are thus negligible. In contrast, changes exceeding 10% are likely to be perceptible to consumers and can potentially impact product acceptability, making them critical for determining shelf‐life and optimizing formulations (Lawless and Heymann 2010; Rosenthal 1999).

**TABLE 3:** Color analysis results in fondant samples.

Extract ratio	L*	a*	b*	∆E*
0%	88.99 ± 0.23^a^	−0.23 ± 0.01^a^	6.46 ± 0.07^a^	—
0.31%	60.05 ± 0.40^b^	−4.42 ± 0.05^b^	18.62 ± 0.29^b^	31.67 ± 4.61^a^
0.63%	55.12 ± 0.59^c^	−4.62 ± 0.04^c^	19.78 ± 0.31^c^	36.65 ± 4.88^b^
1.25%	49.04 ± 0.12^d^	−3.85 ± 0.04^d^	16.57 ± 0.31^d^	41.36 ± 3.52^c^

*Note:* Values are given as mean ± standard deviation. Different letters in the same column indicate a statistically significant difference (*p* < 0.05).

**TABLE 4:** Color analysis values for fondant samples after 1 month storage.

Extract ratio	L*	a*	b*	∆E*
0%	80.22 ± 0.91^a^	−0.23 ± 0.03^a^	7.49 ± 0.98^a^	—
0.31%	64.53 ± 0.82^b^	−4.48 ± 0.12^b^	17.86 ± 0.36^b^	19.27 ± 2.29^a^
0.63%	60.42 ± 0.51^c^	−3.64 ± 0.09^c^	15.05 ± 0.23^c^	21.47 ± 3.37^ab^
1.25%	58.70 ± 0.81^c^	−2.53 ± 0.14^d^	13.07 ± 0.23^d^	22.35 ± 4.28^b^

*Note:* Values are given as mean ± standard deviation. Different letters in the same column indicate a statistically significant difference (*p* < 0.05).

**TABLE 5:** Percentage change rate in color values of fondant samples after 1 month storage.

Extract ratio	L*	a*	b*
0%	9.86%	0.46%	15.96%
0.31%	7.47%	1.30%	4.10%
0.63%	9.60%	21.08%	23.93%
1.25%	19.70%	34.20%	21.13%

*Ulva lactuca* extract significantly altered meringue color properties (*p* < 0.05). Increasing concentrations progressively reduced lightness (L*: 94.40 ± 0.27 control → 66.22 ± 1.77 at 0.63%), intensified green dominance (a*↓), and enhanced yellowness (b*↑), reflecting the extract's chlorophyll and carotenoid profile. Total color difference (ΔE*) increased proportionally with concentration, peaking at 0.63% *Ulva*. Post‐storage, all samples exhibited elevated L* values (e.g., 66.22 ± 1.77 → 78.22 ± 0.25 at 0.63%), likely from pigment degradation or physicochemical changes (e.g., moisture loss, crystallization). Concurrently, green hues intensified (a*↓) while yellowness declined (b*↓), suggesting carotenoid oxidation or interactions with Maillard reaction intermediates. Despite shifts, *Ulva*‐fortified samples retained distinct chromatic profiles versus controls, demonstrating partial pigment stability.

Mechanistically, color evolution in fortified meringues involves dual contributions: direct pigmentation from chlorophylls and carotenoids, and thermally driven interactions between *Ulva* bioactives and Maillard reaction products (e.g., Strecker aldehydes) (Han et al. 2024). Such interactions may stabilize or degrade chromophores, depending on processing conditions. Storage‐induced oxidation and structural changes (e.g., moisture redistribution) further modulate L* and chromatic intensity (Wijesekara and Xu 2024). For instance, chlorophylls demonstrated relative stability, sustaining green hues (a*↓), while carotenoid degradation likely drove yellowness loss (b*↓).

Furthermore, the enhanced stability of the green parameter (a*) observed in the meringue samples compared to the fondant may be attributed to the protective role of the egg white protein network. It is hypothesized that during the formation of the aerated structure, proteins may contribute to the protection of the chlorophyll pigments. In contrast, the sucrose‐rich environment of the fondant lacks such a protective protein‐based framework, leaving the pigments more susceptible to environmental degradation.

Commercial viability of *Ulva lactuca* as a natural colorant hinges on mitigating concentration‐dependent instability. While higher extract loads enhance chromatic intensity, they exacerbate ΔE* deviations during storage. Formulation strategies, such as limiting concentrations to ≤ 0.31%, incorporating antioxidants (e.g., tocopherols), or employing encapsulation, could stabilize pigments without compromising sensory appeal. These approaches align with functional food design principles, emphasizing the integration of natural colorants into aerated matrices while managing structural and aesthetic trade‐offs. Color analysis results (initial and post‐storage) and percentage change rates are presented in Tables 6, 7, 8, respectively.

**TABLE 6:** Color analysis results in meringue samples.

Extract ratio	L*	a*	b*	∆E*
0%	94.40 ± 0.27^d^	0.64 ± 0.14^d^	6.29 ± 0.70^a^	—
0.08%	89.76 ± 0.23^c^	−2.49 ± 0.05^c^	12.76 ± 0.19^b^	8.57 ± 0.73^a^
0.16%	84.45 ± 0.48^b^	−3.06 ± 0.22^b^	18.52 ± 0.38^c^	16.20 ± 1.09^b^
0.31%	84.03 ± 0.58^b^	−3.95 ± 0.12^a^	19.41 ± 0.31^d^	17.36 ± 0.69^b^
0.63%	66.22 ± 1.77^a^	−4.01 ± 0.75^a^	19.89 ± 0.62^d^	31.68 ± 1.48^c^

*Note:* Values are given as mean ± standard deviation. Different letters in the same column indicate a statistically significant difference (*p* < 0.05).

**TABLE 7:** Color analysis values for meringue samples after 1 month storage.

Extract ratio	L*	a*	b*	∆E*
0%	100.19 ± 0.14^a^	0.77 ± 0.06^a^	7.38 ± 0.19^a^	—
0.08%	98.59 ± 0.24^b^	−2.60 ± 0.14^b^	11.49 ± 0.14^b^	5.57 ± 0.18^a^
0.16%	95.23 ± 0.08^c^	−2.94 ± 0.45^c^	14.90 ± 0.58^c^	9.77 ± 0.34^b^
0.31%	89.34 ± 0.18^d^	−3.96 ± 0.08^d^	17.59 ± 0.35^d^	15.65 ± 0.28^c^
0.63%	78.22 ± 0.25^e^	−4.64 ± 0.31^e^	18.72 ± 0.19^e^	25.35 ± 0.85^d^

*Note:* Values are given as mean ± standard deviation. Different letters in the same column indicate a statistically significant difference (*p* < 0.05).

**TABLE 8:** Percentage change rate in color values of meringue samples after 1 month storage.

Extract ratio	L*	a*	b*
0%	5.80%	16.88%	14.77%
0.08%	8.96%	4.23%	11.05%
0.16%	11.32%	4.08%	24.30%
0.31%	5.94%	0.25%	10.35%
0.63%	15.34%	13.58%	6.25%

The observed differences in pigment stability and overall performance between the two matrices are fundamentally driven by distinct matrix–compound interactions. In the aerated meringue system, alga‐protein interactions play a critical role. Amphiphilic extract components and polar phenolics interact with egg white proteins (e.g., ovalbumin) primarily via non‐covalent bonds, such as hydrogen bonding and hydrophobic interactions. While these interactions can partially protect bioactives during thermal processing, they also compete with the necessary protein–protein interfacial bonds, leading to foam destabilization, altered light scattering, and increased chromatic degradation (higher Δ*E*) during storage. Conversely, in the fondant system, alga‐sucrose interactions govern the matrix behavior. The extract's hydroxyl‐rich compounds (phenolics and sulfated polysaccharides) form extensive hydrogen bonds with the dense sucrose network. This traps free water (lowering water activity) but also disrupts normal sugar crystallization, causing the observed concentration‐dependent softening. Furthermore, the dense, low‐moisture sucrose matrix acts as a superior physical barrier against oxygen diffusion compared to the highly porous meringue, which explains the better preservation of chlorophyll and carotenoid pigments (a* and b* stability) in fondant during storage.

### 3.2 Dry Matter Content and Water Activity

Fondant dry matter content increased linearly with *Ulva* extract concentration (Table 9), rising from 90.71% ± 0.00% (control) to 92.66% ± 0.01% (1.25% extract). This is attributed to the post‐cooking addition of extract, which preserved heat‐sensitive compounds relatively intact and introduced non‐volatile solids (e.g., phenolics, pigments), likely contributed to the observed increase in dry matter content, and thereby elevating total dry matter proportionally to extract dosage. Water activity (*a*_w_) decreased significantly (*p* < 0.05) from 0.81 ± 0.00 (control) to 0.70 ± 0.00 (1.25% extract), suggesting *Ulva*'s constituents (e.g., polar molecules like phenolics) act as solutes, binding free water and reducing its availability in the system. The inverse dry matter–*a*_w_ relationship implies *Ulva*'s solids directly modulate water interactions. All *a*_w_ values (control and treated) were consistent with the established fondant range (0.65–0.87) in the literature (Ergun et al. 2010; Plotnikova et al. 2021).

**TABLE 9:** Dry matter and water activity values for fondant samples.

Extract ratio	Dry matter (%)	*a*_w_
0%	90.71 ± 0.00^a^	0.81 ± 0.00^a^
0.31%	91.81 ± 0.00^ab^	0.74 ± 0.00^b^
0.63%	92.02 ± 0.00^b^	0.72 ± 0.00^c^
1.25%	92.66 ± 0.01^b^	0.70 ± 0.00^d^

*Note:* Values are given as mean ± standard deviation. Different letters in the same column indicate a statistically significant difference (*p* < 0.05).

In contrast, meringue incorporated *Ulva* extract before baking. Dry matter content decreased with increasing extract concentration (Table 10): control (99.49% ± 0.03%) > 0.63% extract (99.10% ± 0.05%). Concentrations of 0.08% and 0.16% had no significant effect (*p* > 0.05) versus control. This lack of statistical significance at lower inclusion levels is rationally expected. Because the baked meringue inherently possesses an extremely high dry matter content (~99.5%), the physical addition of microscopic extract masses (0.08% and 0.16%) contributes a negligible amount of total solids. This minimal variation falls within the standard deviation of the gravimetric measurement and is easily normalized by the extensive water evaporation during the baking process. Duncan's test confirmed significant differences (*p* < 0.05) between higher concentrations. This decline may be explained by thermal degradation of thermolabile compounds (e.g., phenolics, pigments) during baking. This thermal degradation may have reduced the final amount of solid bioactive material in the product, resulting in a lower dry matter percentage as extract concentration increased (Blanch and Ruiz del Castillo 2021). Additionally, the nature of the meringue matrix—aerated and porous—may have further facilitated the loss of volatiles or moisture‐binding interactions, influencing the dry matter outcome. In contrast, the denser structure of fondant may have allowed for better retention of these compounds, especially when added after thermal treatment (Guichard 2015). Despite lower dry matter, *a*_w_ decreased significantly (*p* < 0.05) from 0.45 ± 0.01 (control) to 0.30 ± 0.01 (0.63% extract). This reduction may arise from the hygroscopic properties of *Ulva* extract, binding free water within the matrix, thus reducing the availability of unbound water and thereby lowering *a*_w_. Duncan's multiple range test confirmed statistically significant differences (*p* < 0.05) in *a*_w_ between specific *Ulva* concentrations, reinforcing the concentration‐dependent effect. The results of the dry matter content and water activity analyses conducted on meringue samples are provided in Table 10.

**TABLE 10:** Dry matter and water activity values for meringue samples.

Extract ratio	Dry matter (%)	*a*_w_
0%	99.49 ± 0.03^a^	0.45 ± 0.01^a^
0.08%	99.48 ± 0.04^a^	0.41 ± 0.01^b^
0.16%	99.48 ± 0.01^a^	0.31 ± 0.01^c^
0.31%	99.27 ± 0.01^b^	0.30 ± 0.02^c^
0.63%	99.10 ± 0.05^c^	0.30 ± 0.01^c^

*Note:* Values are given as mean ± standard deviation. Different letters in the same column indicate a statistically significant difference (*p* < 0.05).

### 3.3 Total Phenolic Content in Sample Extracts

**FIGURE 3:**
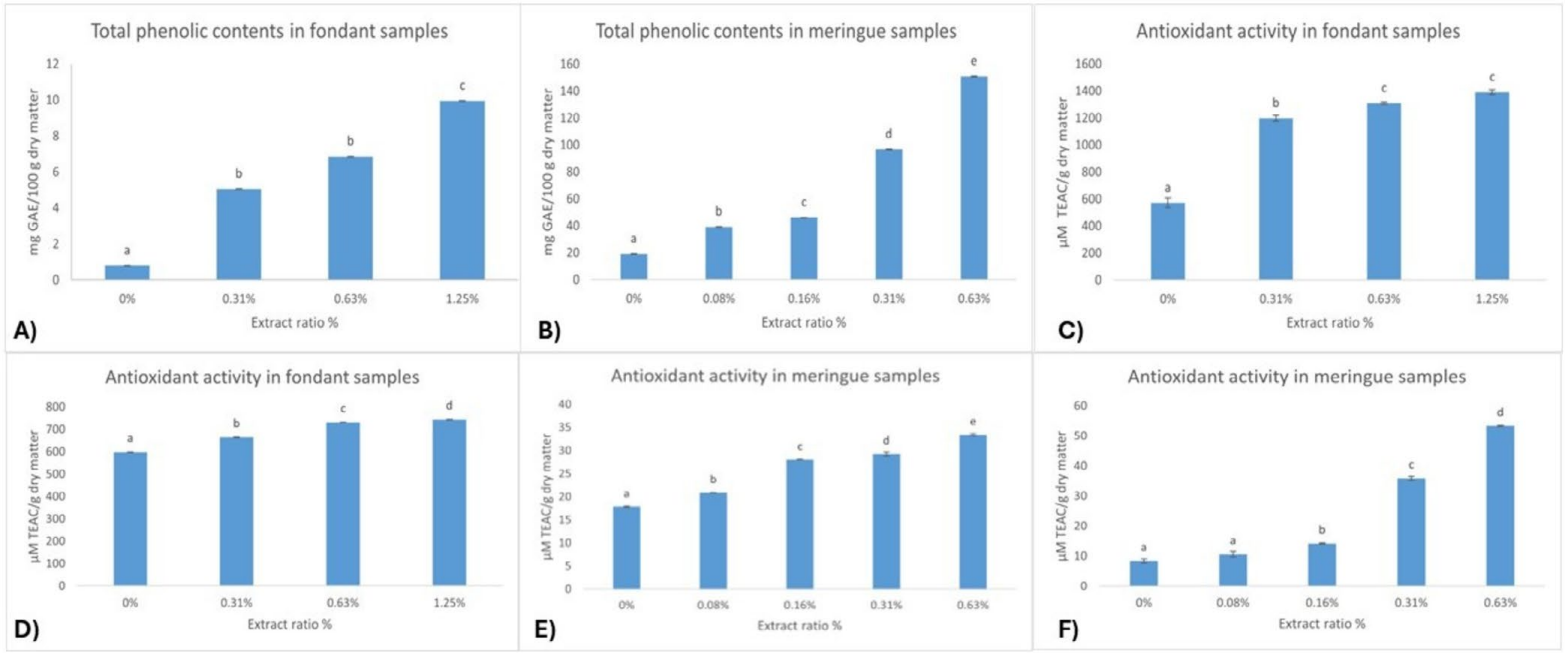
Total phenolic contents and antioxidant activities of the samples. (A) Total phenolic contents in fondant, (B) Total phenolic contents in meringue, (C) Antioxidant activity of fondant determined by the ABTS assay, (D) Antioxidant activity of fondant determined by the DPPH assay, (E) Antioxidant activity of meringue determined by the ABTS assay, (F) Antioxidant activity of meringue determined by the DPPH assay. Values are expressed as mg GAE/100 g dry sample for total phenolic contents and μM Trolox equivalents (TEAC/g dry matter) for antioxidant activities. Error bars represent standard deviations (*n* = 3).

Total phenolic content (TPC) in fondant samples increased proportionally with *Ulva* extract concentration (0%–1.25% w/w). The control showed minimal TPC (0.80 ± 0.02 mg GAE/100 g DM), consistent with its primary composition of sucrose. Extract incorporation significantly increased TPC concentration‐dependently: 0.31% yielded 5.06 ± 0.14 mg GAE/100 g DM. A significant rise (*p* < 0.05) occurred between 0% and 0.31%, plateauing at 0.31%–0.63% (*p* > 0.05), followed by further elevation at 1.25% (*p* < 0.05). This demonstrates a strong positive linear relationship between extract ratio and TPC enrichment without saturation. The data confirm the extract as a promising phenolic source for fondant fortification (Figure 3A). Minimal thermal degradation during preparation likely preserved heat‐sensitive phenolics, affirming the extract's efficacy as a functional enhancer.

Meringues fortified with *Ulva* extract exhibited a ≈7‐fold TPC increase (19.13 ± 0.04 → 150.95 ± 0.15 mg GAE/100 g) at 0.63% extract, with Duncan's test confirming significant differences (*p* < 0.05) across concentrations (Figure 3B). These results align with studies linking phenolic abundance to radical‐scavenging potential, as seen in *Ulva lactuca* and *Ulva fasciata* , which are rich in phenolics and flavonoids (Tanna et al. 2019).

Comparative studies highlight formulation and processing impacts on phenolic retention. Crackers with 4% *Ulva lactuca* biomass showed marginal TPC increases (0.47 to 0.50 mg GAE/g DM; *p* > 0.05), whereas biscuits with 5% *Caulerpa racemosa* biomass achieved significant TPC elevation (*p* < 0.05), attributed to lower baking temperatures (190°C) and higher algal incorporation (Kumar et al. 2018; Egodavitharana et al. 2023). Such disparities underscore the need to optimize concentration and thermal parameters when using whole biomass versus extracts. Species‐specific variations further influence phenolic enrichment: *Himanthalia elongata* (17.07% w/w in crackers) elevated TPC to 138.25 mg GAE/100 g DM, while wakame‐enriched pasta (70:30 semolina/wakame) achieved 0.94 mg GAE/g (Cox and Abu‐Ghannam 2013; Prabhasankar et al. 2009). Microalgae like *Arthrospira platensis* (6% in cookies) yielded 0.90 mg GAE/g, outperforming chlorophyte species (Batista et al. 2017). These findings emphasize that tailored algal selection and processing strategies are essential to maximize phenolic delivery in fortified foods. Consistent with this study, *Ulva* extracts offer a sustainable, concentration‐dependent means of enhancing phenolic content in confectionery matrices.

The significant TPC discrepancy between *Ulva*‐fortified fondant (0.80–9.96 mg GAE/100 g dry sample) and meringue (19.13–150.95 mg GAE/100 g dry sample) is a clear observed finding. Existing literature suggests it may be influenced by several interrelated factors: the timing of extract incorporation, the distinct physicochemical properties of the matrices, and theoretical interactions between phenolic compounds and other food components (Rocchetti et al. 2022). For instance, in fondant, post‐thermal extract addition might limit phenolic‐matrix interactions, potentially yielding lower TPC. Conversely, literature indicates that pre‐baking addition in meringue could enhance bound phenolic release or extractability during thermal processing (Minatel et al. 2017). Furthermore, it is hypothesized that meringue's egg white proteins could stabilize phenolics via non‐covalent bonds (e.g., hydrogen bonding, hydrophobic interactions), theoretically protecting them during baking and enhancing extractability during analysis (Kieserling et al. 2024). In contrast, fondant's sugar‐rich, dense structure is thought to limit the diffusion of phenolic compounds (Schefer et al. 2021). Additionally, because the Folin–Ciocalteu assay is susceptible to interference, fondant's high reducing sugar content may theoretically underestimate phenolic content by masking phenolics (Sánchez‐Rangel et al. 2013). Therefore while the higher TPC in the meringue matrix is a clearly observed finding, the specific mechanisms driving this difference remain theoretical and warrant further experimental investigation.

### 3.4 Antioxidant Activity by DPPH and ABTS Assays

Recent studies have demonstrated the antioxidant potential of algal bioactives in diverse food applications. For example, *Arthrospira platensis* (spirulina) and *Chlorella vulgaris* have been successfully incorporated into baked goods to enhance antioxidant capacity, while *Himanthalia elongata* (brown seaweed) improved phenolic content in fortified crackers (Batista et al. 2019; Cox and Abu‐Ghannam 2013).

*Ulva lactuca* extract significantly increased antioxidant activity in fondant and meringue matrices. In fondant, antioxidant activity rose proportionally with extract ratio, from 571.30 ± 37.10 μM TEAC/g dry matter (0% *Ulva*) to 1391.67 ± 18.54 μM TEAC/g dry matter (1.25% *Ulva*). The steep ≈2.1‐fold increase between 0% and 0.31% *Ulva* suggests a threshold effect where minimal incorporation significantly enhances radical scavenging. Activity plateaued slightly at higher concentrations (0.63%–1.25%), indicating potential saturation in ABTS‐reactive antioxidant interactions. DPPH assays confirmed a gradual dose‐dependent increase, from 598.31 ± 0.76 μM TEAC/g dry matter (0% *Ulva*) to 744.68 ± 0.12 μM TEAC/g dry matter (1.25% *Ulva*). The smaller magnitude of change (≈1.2‐fold) compared to ABTS implies *Ulva*'s antioxidants exhibit greater affinity for ABTS^+^ radicals, likely due to differences in polarity or steric accessibility between assays. Minimal DPPH standard deviations (±0.08–1.02) reflect high reproducibility. Fondant antioxidant activity results are presented in Figure 3C,D for ABTS and DPPH assays, respectively.

For meringue samples, ABTS activity rose steadily from 17.84 ± 0.13 μM TEAC/g dry matter (0% *Ulva*) to 33.42 ± 0.26 μM TEAC/g dry matter (0.63% *Ulva*), representing a 1.9‐fold enhancement. This linear increase (≈4.5 μM TEAC/g dry matter per 0.1% *Ulva*), with no saturation, suggests ABTS's sensitivity to hydrophilic antioxidants like polar phenolics or flavonoids. Conversely, DPPH activity showed a more pronounced, nonlinear surge, escalating from 8.37 ± 0.76 μM TEAC/g dry matter (0% *Ulva*) to 53.28 ± 0.24 μM TEAC/g dry matter (0.63% *Ulva*)—a 6.37‐fold increase. The sharp rise at ≥ 0.31% *Ulva* indicates a threshold effect where higher concentrations enhance scavenging efficiency against lipophilic DPPH radicals, likely due to the dominance of lipophilic antioxidants (e.g., carotenoids, chlorophyll derivatives) at elevated doses. Meringue antioxidant activity results are presented in Figure 3E,F for ABTS and DPPH assays, respectively.

The substantial discrepancy in absolute antioxidant values between the fondant and meringue systems may be attributed to their distinct processing conditions and baseline matrix properties. Furthermore, as detailed in the methodology, the *Ulva* extract was incorporated into the fondant post‐thermal treatment, thereby preserving thermolabile antioxidant compounds. In contrast, the extract was incorporated into the meringue prior to baking, which may cause these sensitive bioactives to high oven temperatures that result in thermal degradation.

The differential sensitivity between ABTS and DPPH assays observed in the *Ulva*‐fortified matrices can be attributed to the differing solubility and reaction mechanisms of the radicals. The ABTS radical is soluble in both aqueous and organic solvents, making it highly applicable to evaluate both hydrophilic and lipophilic antioxidant systems (Floegel et al. 2011). Conversely, the DPPH radical is primarily dissolved in organic media, restricting its sensitivity mostly to hydrophobic compounds. Therefore, the simultaneous use of both assays provides complementary insights. While the ABTS assay offers a broader estimation of the total antioxidant capacity (capturing polar phenolics and sulfated polysaccharides), the DPPH assay specifically highlights the radical‐scavenging contribution of the lipophilic constituents, such as carotenoids and chlorophyll derivatives. Together, these assays confirm that the antioxidant efficacy of *Ulva lactuca* extract relies on a complex synergy between its water‐soluble and fat‐soluble bioactives.

These findings align with prior findings on *Ulva rigida* (IC_50_: 3.76 mg/g for DPPH) and solvent‐dependent antioxidant extraction from *Ulva lactuca* (methanol extract: 79.63% inhibition) (Ak and Türker 2019; Çebi et al. 2016). The dual functionality of *Ulva* as an antioxidant and preservative, evidenced by reduced lipid oxidation in meat products (Orhan 2019), further supports its potential in confectionery applications. Collectively, these findings affirm *Ulva* as a sustainable, multifunctional ingredient for enhancing oxidative stability in diverse food systems.

### 3.5 Baking Yield

Analysis revealed a consistent, significant decline (*p* < 0.05) in meringue baking yield with *Ulva* extract addition versus control. The findings indicate that baking yield was significantly affected (*p* < 0.05) by the addition of the extract (Table 11). This phenomenon is hypothesized to arise from the denaturation of egg white proteins by phenolic compounds in the extract, reducing foam stability. The compromised foam facilitates air cell collapse, amplifying moisture and air loss during baking (Wouters et al. 2018). Phenolic‐induced protein precipitation may also create heterogeneous voids in the matrix, promoting liquid leakage. Structural collapse further increases surface area, accelerating evaporative losses. Collectively, these mechanisms likely contribute to reduced baking efficiency in extract‐incorporated meringues (Hu and Meng 2023).

**TABLE 11:** Baking yield values in meringue samples.

Extract ratio	*W*_sample_ (g)	*W*_foam_ (g)	Baking yield (%)
0%	8.27	10.70	77.25 ± 0.24^a^
0.08%	7.97	10.53	75.65 ± 0.36^b^
0.16%	7.97	10.97	72.66 ± 0.35^c^
0.31%	7.50	10.50	71.44 ± 0.16^d^
0.63%	7.50	10.77	69.66 ± 0.30^e^

*Note:* Values are given as mean ± standard deviation. Different letters in the same column indicate a statistically significant difference (*p* < 0.05).

### 3.6 Volume Index

Analysis revealed a significant decline (*p* < 0.05) in meringue volume index versus the control. Samples with 0.08% and 0.16% *Ulva* extract showed slight differences in between, while higher concentrations (0.31% and 0.63%) caused significant reductions (*p* < 0.05) (Table 12). This trend may be attributed to the disruption of the egg white protein network, diminishing the ability of air cells to expand and stabilize. Polar components in the extract also may have increased foam viscosity, limiting air incorporation during whipping (Wouters et al. 2018). Pre‐baking foam collapse at high extract concentrations likely impeded oven spring, resulting in samples deviating from the traditional meringue structure (Froning et al. 1981). These findings demonstrate the concentration‐dependent impact of *Ulva* extract on aerated matrices, underscoring the need to balance functional enrichment with structural integrity in confectionery.

**TABLE 12:** Volume index values in meringue samples.

Extract ratio	*B*	*C*	*D*	Volume index
0%	23.62	12.80	16.85	319.03 ± 6.04^a^
0.08%	19.60	13.29	17.00	277.61 ± 4.63^b^
0.16%	19.85	12.80	14.99	268.74 ± 11.53^b^
0.31%	13.30	14.50	15.20	207.29 ± 10.07^c^
0.63%	5.52	13.29	15.10	88.43 ± 5.84^d^

*Note:* Values are given as mean ± standard deviation. Different letters in the same column indicate a statistically significant difference (*p* < 0.05).

### 3.7 Texture Profile Analysis

Instrumental texture analysis revealed that incorporating *Ulva* extract induced statistically significant alterations (*p* < 0.05) across all primary texture parameters in both confectionery formulations (Tang et al. 2017).

For the fondant matrices (Table 13), increasing *Ulva* extract ratios induced distinct structural alterations (Szczesniak 2002). At 0.31%, hardness slightly increased, suggesting a temporary structural stabilization. However, higher ratios (0.63% and 1.25%) caused a progressive decline in hardness (250 g → 235 g) and adhesiveness (−120.11 → −95.08 g·s), reflecting disrupted sucrose crystallization and reduced surface tackiness (Ozcan et al. 2019). Concurrently, springiness, cohesiveness, gumminess, chewiness, and resilience all decreased in a concentration‐dependent manner. These trends indicate that the extract's bioactives and pigments likely act as filler particles and disrupt hydrogen bonding, thereby weakening the sugar network's continuous structure and elastic recovery capacity (Le Bourvellec and Renard 2012).

**TABLE 13:** Textural properties of fondant samples.

Extract ratio	Hardness (g)	Adhesiveness (g·s)	Springiness	Cohesiveness	Gumminess	Chewiness	Resilience
0%	260.16 ± 10^b^	−120.11 ± 10^a^	0.82 ± 0.03^c^	0.55 ± 0.02^c^	143.14 ± 6^c^	117.32 ± 5^c^	0.32 ± 0.02^c^
0.31%	270.04 ± 12^c^	−110.16 ± 9^ab^	0.79 ± 0.03^bc^	0.53 ± 0.02^bc^	143.01 ± 7^c^	113.06 ± 6^c^	0.31 ± 0.02^bc^
0.63%	250.18 ± 8^ab^	−100.18 ± 9^b^	0.74 ± 0.03^b^	0.50 ± 0.02^bc^	125.16 ± 6^b^	93.36 ± 4^b^	0.28 ± 0.02^ab^
1.25%	235.43 ± 9^a^	−95.08 ± 8^b^	0.68 ± 0.03^a^	0.46 ± 0.02^a^	108.07 ± 6^a^	73.14 ± 4^a^	0.26 ± 0.02^a^

*Note:* Values are given as mean ± standard deviation. Different letters in the same column indicate a statistically significant difference (*p* < 0.05).

In the meringue samples (Table 14), while hardness remained statistically stable up to 0.31%, the 0.63% extract sample exhibited a sharp reduction (963.39 ± 13.54 g) compared to the control (1776.06 ± 69.56 g). This profound softening is attributed to the interaction between algal phenolics and egg white proteins (e.g., ovalbumin), which disrupts critical hydrogen bonds and disulfide bridges, destabilizing the aerated foam structure (Diaz et al. 2022). Similar reductions in firmness by structural and algal additives have been well‐documented in other aerated or baked matrices (Shyu and Sung 2010; Onacık‐Gür et al. 2018). Furthermore, lipophilic carotenoids create heterogeneous microdomains within the fat‐free matrix, while hydrophilic components retain moisture, shifting the texture from a dry, brittle state to a softer, plastically deformable structure (Wouters et al. 2018; Salehi and Kashaninejad 2017).

**TABLE 14:** Textural properties of meringue samples.

Extract ratio	Hardness (g)	Fracturability (g)	Adhesiveness (g·s)	Springiness	Cohesiveness	Gumminess (g)	Chewiness (g)	Resilience
0%	1776.06 ± 70^c^	2934.65 ± 16^e^	−0.94 ± 0.22^a^	0.90 ± 0.03^c^	0.55 ± 0.02^d^	976.06 ± 38^e^	878.14 ± 35^e^	0.36 ± 0.04^d^
0.08%	1425.02 ± 314^b^	2416.82 ± 26^d^	−0.85 ± 0.10^ab^	0.88 ± 0.04^c^	0.52 ± 0.02^cd^	741.34 ± 33^d^	652.03 ± 29^d^	0.33 ± 0.03^cd^
0.16%	1348.95 ± 3^b^	2343.69 ± 29^c^	−0.66 ± 0.05^bc^	0.86 ± 0.02^bc^	0.49 ± 0.01^c^	661.03 ± 12^c^	568.04 ± 10^c^	0.30 ± 0.03^bc^
0.31%	1257.63 ± 30^b^	2125.61 ± 19^b^	−0.54 ± 0.04^cd^	0.82 ± 0.02^ab^	0.45 ± 0.02^b^	566.01 ± 26^b^	464.11 ± 22^b^	0.27 ± 0.02^ab^
0.63%	963.39 ± 14^a^	1599.53 ± 30^a^	−0.34 ± 0.03^d^	0.78 ± 0.03^a^	0.40 ± 0.02^a^	384.66 ± 17^a^	300.41 ± 16^a^	0.22 ± 0.03^a^

*Note:* Values are given as mean ± standard deviation. Different letters in the same column indicate a statistically significant difference (*p* < 0.05).

Consequently, the compromised protein network and altered water‐binding capacity led to parallel decreases in meringue adhesiveness, fracturability, elasticity, and cohesiveness (Hu and Meng 2023; Narsimhan and Xiang 2018; van de Velde et al. 2015; Shahbazizadeh et al. 2015). Secondary parameters like gumminess, chewiness, and resilience also diminished progressively as the structural integrity weakened (Lee et al. 2015; Zhan et al. 2022).

Based on these findings, determining the precise incorporation threshold is pivotal. For meringues, concentrations of 0.08%–0.16% represent the optimal Ulva extract range. This specific ratio successfully mitigates severe protein network disruption and moisture‐induced softening, ensuring that the functional enrichment aligns with sensory expectations by preserving the essential crunchy and aerated structural integrity (Wouters et al. 2018; Diaz et al. 2022).

### 3.8 Sensory Evaluation

Sensory evaluation (Tables 15 and 16) revealed that *Ulva lactuca* extract significantly affected all parameters (appearance, texture, odor, overall acceptability) in both matrices (*p* < 0.05). As expected, increasing extract concentrations led to a consistent, dose‐dependent decline in sensory scores. For both fondant and meringue, the control samples achieved the highest ratings, while the maximum extract concentrations exhibited the most marked deterioration.

**TABLE 15:** Sensory analysis results of fondant samples.

Sample code	Extract ratio	Appearance	Texture	Odor	Overall acceptability
F‐1	0%	8.80 ± 0.04^d^	8.70 ± 0.04^d^	8.62 ± 0.04^d^	8.49 ± 0.06^d^
F‐2	0.31%	7.89 ± 0.03^c^	7.63 ± 0.04^c^	7.51 ± 0.04^c^	7.40 ± 0.05^c^
F‐3	0.63%	6.97 ± 0.06^b^	6.71 ± 0.03^b^	6.41 ± 0.03^b^	6.30 ± 0.04^b^
F‐4	1.25%	6.10 ± 0.05^a^	5.80 ± 0.06^a^	5.62 ± 0.05^a^	5.39 ± 0.04^a^

*Note:* Values are given as mean ± standard deviation. Different letters in the same column indicate a statistically significant difference (*p* < 0.05).

**TABLE 16:** Sensory analysis results of meringue samples.

Sample code	Extract ratio	Appearance	Texture	Odor	Overall acceptability
M‐1	0%	8.73 ± 0.26^c^	8.53 ± 0.14^c^	8.47 ± 0.21^d^	8.53 ± 0.22^c^
M‐2	0.08%	8.16 ± 0.32^c^	7.77 ± 0.59^b^	8.03 ± 0.18^c^	8.10 ± 0.50^c^
M‐3	0.16%	7.43 ± 0.16^b^	7.40 ± 0.47^b^	7.37 ± 0.40^c^	8.02 ± 0.37^c^
M‐4	0.31%	7.17 ± 0.06^ab^	7.13 ± 0.16^b^	7.01 ± 0.32^b^	7.17 ± 0.88^b^
M‐5	0.63%	6.60 ± 0.55^a^	6.23 ± 0.41^a^	6.17 ± 0.68^a^	6.33 ± 0.98^a^

*Note:* Values are given as mean ± standard deviation. Different letters in the same column indicate a statistically significant difference (*p* < 0.05).

The reduction in sensory scores across both matrices can be attributed to the inherent biochemical properties of the alga. Appearance scores declined primarily due to the intense green pigmentation of chlorophylls and xanthophylls, which exceeded visual tolerance at elevated levels (Martins et al. 2021). Odor acceptability dropped significantly as marine and earthy volatiles from the extract clashed with the inherent sweetness of the confections (Mouritsen et al. 2018b). Furthermore, texture scores were negatively impacted by the extract's moisture retention and polysaccharides; these components disrupted the normal crystallization in the fondant matrix and compromised the delicate, aerated protein network in the meringues, causing a loss of their characteristic crispiness (Mouritsen et al. 2018a; Wouters et al. 2018; Froning et al. 1981).

Despite these declines, optimal incorporation thresholds were identified where sensory compromise is minimized. For fondant, the 0.31% extract sample maintained acceptable overall scores (> 7.0), indicating a successful balance between functionality and palatability. Meringues exhibited greater structural and sensory sensitivity, maintaining resilience at concentrations ≤ 0.16% but deteriorating sharply at ≥ 0.31% *Ulva*.

To overcome the sensory limitations at higher incorporation levels, future studies could explore flavor‐masking strategies using compatible natural additives, such as vanilla or citrus extracts, which could neutralize the earthy and marine notes without compromising the product's clean‐label status.

Ultimately, these findings underscore that *Ulva* extract can be successfully incorporated up to 0.31% in fondant and 0.16% in meringue, but precise dose selection is critical to balance bioactive enrichment with overall consumer satisfaction.

### 3.9 Structural Characterization via FT‐IR Spectroscopy

**FIGURE 4:**
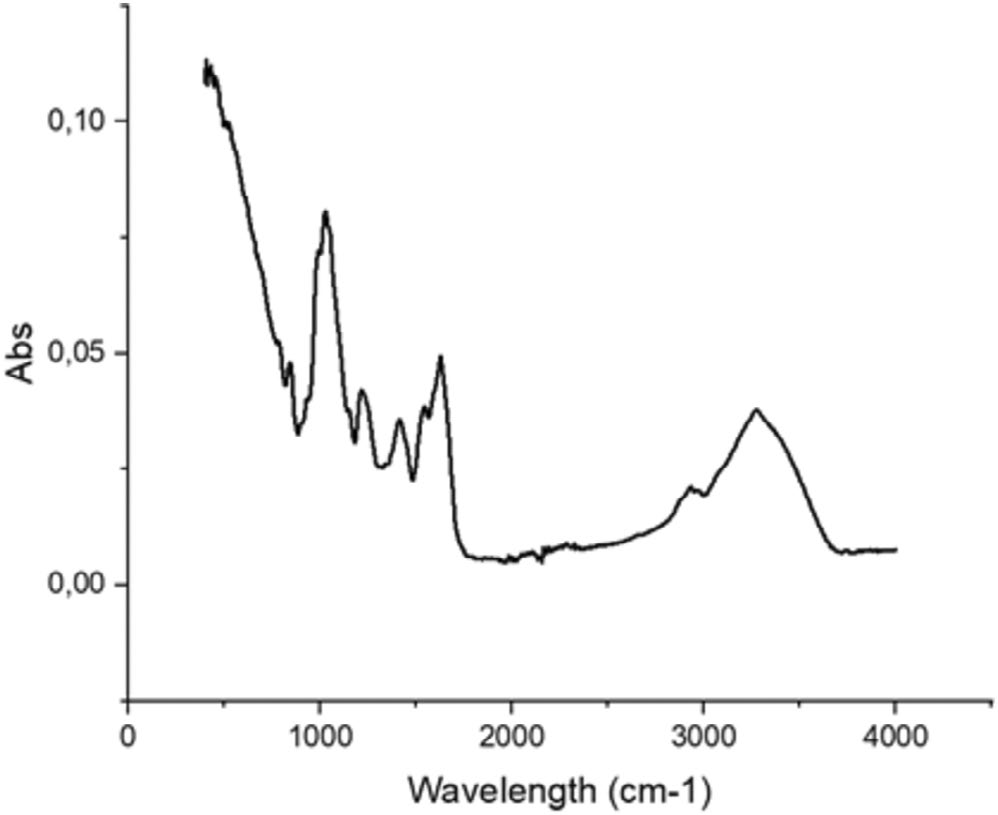
FT‐IR spectra of *Ulva lactuca* .

Fourier‐transform infrared (FT‐IR) spectroscopy was utilized to specifically identify the diverse bioactive functional groups (such as phenolics and sulfated polysaccharides) within the dried *Ulva lactuca* samples (Figure 4). The broad band at 3300–3400 cm^−1^ corresponds to —OH stretching vibrations, reflecting the abundance of hydroxyl groups in ulvan, including both polysaccharide backbones and phenolic moieties (Lahaye and Robic 2007). These functional groups are closely associated with the antioxidant activity confirmed by DPPH and ABTS assays.

The weak band around 2900 cm^−1^ reflects aliphatic C—H stretching of methylene and methyl groups, while the strong peak near 1650 cm^−1^ is linked to carboxylate (COO^−^) asymmetric stretching, characteristic of *Ulva* sp. (Yan et al. 2019). Notably, the distinct peak at ~1250 cm^−1^ corresponds to S=O stretching of sulfate esters, confirming the presence of sulfated polysaccharides typical of *Ulva* extracts (Yaich et al. 2017).

Pronounced bands between 1000 and 1200 cm^−1^, corresponding to C—O and C—O—C stretching vibrations, indicate the presence of monosaccharides like rhamnose and glucuronic acid in the *Ulva* matrix (Robic et al. 2009). Additionally, bands at 850–790 cm^−1^, attributed to sugar ring deformations and sulfate group patterns, reflect ulvan‐derived structural diversity. Overall, these spectral findings characterize the biochemical profile of *Ulva lactuca* biomass and confirm the presence of reactive functional groups (e.g., hydroxyl and sulfate groups), which may provide a theoretical molecular basis for the bioactive and physicochemical changes observed in the confectionery systems following extract incorporation.

### 3.10 Microstructural Analysis Using Scanning Electron Microscopy (SEM)

**FIGURE 5:**
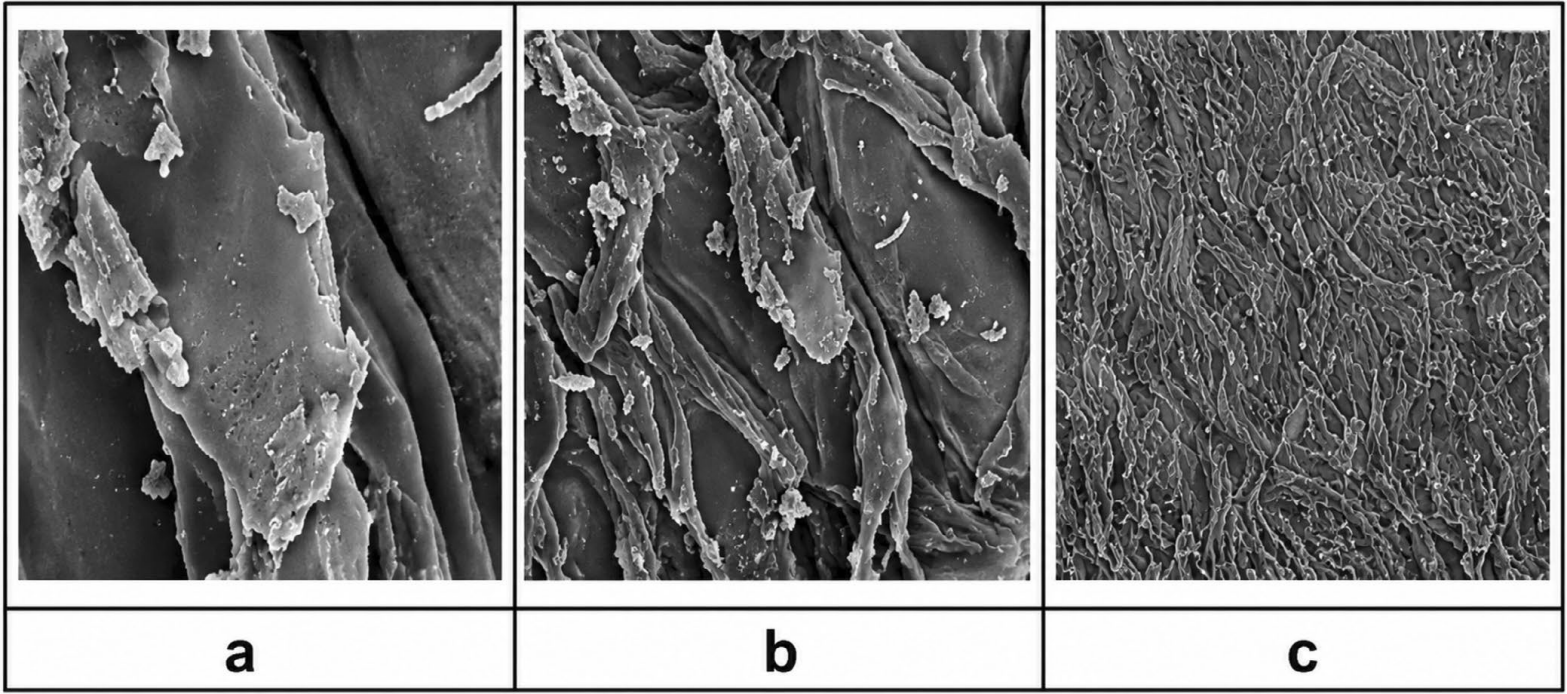
SEM images of *Ulva lactuca* at different zooms: (a) 65,000×, (b) 20,000×, and (c) 2000×.

Scanning electron microscopy (SEM) was employed to characterize the surface morphology and porous microstructure of the raw *Ulva lactuca* , which fundamentally dictates its high extraction efficiency and the subsequent release of bioactive compounds into the final food matrices. Figure 5 displays SEM images of the *Ulva lactuca* sample at varying magnifications. The irregular, wrinkled morphology and high surface area of the amorphous structure suggest advantages in color homogeneity and water retention (Jmel et al. 2019). The fibrous and microporous structure aligns with regions rich in phenolic compounds and sulfated polysaccharides, supporting enhanced antioxidant activity (Ibrahim et al. 2022). DPPH and ABTS results further corroborate the role of this morphology in bioactive delivery.

The fibrous nature of *Ulva* sp. likely contributes to textural changes (e.g., reduced hardness) in sugar matrices via physical interactions with starch‐sugar networks (Yu‐Qing et al. 2016). The presence of —OH and —SO_3_ groups (identified via FT‐IR) may modulate hydrophobic‐hydrophilic balance, influencing water activity in fondant (Robic et al. 2009).

In conclusion, SEM images reveal microstructural features (amorphous surface, porosity, fibrous form) that underpin *Ulva*'s role as a functional additive for color stability, antioxidant efficacy, and texture modulation. These properties position it as a sustainable alternative for natural food coloring and functional food development.

## 4 Conclusion

Addressing its primary objective to develop functional confectionery systems, this study successfully demonstrated the potential of *Ulva lactuca* extract as a sustainable, bioactive ingredient for fondant and meringue. While the maximum tested extract concentrations (1.25% in fondant and 0.63% in meringue) yielded the highest antioxidant capacity, phenolic enrichment, and green pigmentation, they also induced significant sensory and textural deterioration. Consequently, lower incorporation levels (0.31% for fondant and 0.16% for meringue) were identified as the optimal thresholds, successfully balancing the trade‐off between maximizing health‐promoting functionality and maintaining high overall consumer acceptability in terms of color, taste, odor, and texture. To translate these bench‐top findings into commercial production and support the blue economy, evaluating the industrial scalability of ultrasound‐assisted extraction (UAE), such as continuous‐flow systems, will be essential. Furthermore, a key limitation of this study is its primary focus on freshly prepared products; although physical color stability was monitored for one month, algal bioactives and chlorophylls remain highly sensitive to degradation by light and oxygen over time. Therefore, future research must prioritize comprehensive, long‐term shelf‐life studies evaluating chemical and microbiological stability, alongside exploring microencapsulation, flavor‐masking agents (e.g., vanilla or citrus extracts), and molecular‐level ingredient–matrix interactions to fully enhance the applicability of *Ulva lactuca* in clean‐label functional foods.

## Author Contributions

**Buse Ozdere Yilmaz:** methodology, validation, data curation. **Elvan Gizem Gursoy:** methodology, validation, data curation. **Sebnem Tavman:** supervision, conceptualization, methodology, funding acquisition. **Meltem Conk Dalay:** project administration. **Aysegul Erdogan:** investigation.

## Funding

This work was supported by the Scientific and Technological Research Council of Turkey (TUBITAK) (Grant Number: 123Y090).

## Ethics Statement

The authors have nothing to report.

## Consent

Written informed consent was obtained from all study participants.

## Conflicts of Interest

The authors declare no conflicts of interest.

## Data Availability

The datasets generated and analyzed during the current study are available from the corresponding author upon reasonable request.
